# Feasibility of ultrasound-guided capsule-sheath space block combined with anterior cervical cutaneous nerves block for thyroidectomy: an observational pilot study

**DOI:** 10.1186/1471-2253-15-4

**Published:** 2015-01-19

**Authors:** Quanguang Wang, Zhengqian Li, Shihao Xu, Yu Li, Xuezheng Zhang, Qimin Liu, Yun Xia, Thomas J Papadimos, Xuzhong Xu

**Affiliations:** Department of Anesthesiology, First Affiliated Hospital, Wenzhou Medical University, Zhejiang, China; Department of Anesthesiology, Peking University Third Hospital, Beijing, China; Department of Anesthesiology, Yongjia People’s Hospital, Zhejiang, China; Department of Anesthesiology, Ohio State University Medical Center, Ohio, USA

**Keywords:** Regional anesthesia, Ultrasound guidance, Thyroidectomy, Contrast medium, Ropivacaine

## Abstract

**Background:**

We evaluated the efficacy of a new anesthetic technique termed ultrasound-guided capsule-sheath space block (CSSB) combined with anterior cervical cutaneous nerve block (CCNB) for thyroidectomy.

**Methods:**

The study included two parts: Part one was an imaging study to determine technique feasibility. The CSSB was performed on five healthy volunteers by introducing the needle 0.5 cm lateral to the probe under in-plane needle ultrasound guidance. After puncture of the false capsule and its subsequent contraction with the true capsule of thyroid, 10 mL of contrast medium was deposited slowly in the capsule-sheath space. The CCNB was performed bilaterally as follows: Under ultrasound guidance, a subcutaneous injection was made along the sternocleidomastoid using 10 mL of contrast medium which was followed by a girdle-shaped picchu raised from the cricoid cartilage to supraclavicular region. The spreading pattern of contrast medium was imaged using computed tomographic scanning. In part two (a clinical case series) the technique efficacy was evaluated. Seventy-eight patients undergoing thyroidectomy had ultrasound-guided CSSB and CCNB with local anesthetics. The sensory onset of CCNB, intraoperative hemodynamic parameters, and analgesic effect were assessed and complications were noted.

**Results:**

The distribution of contrast medium was well defined. In part two the onset time of CCNB was 2.2 ± 0.7 min, and the hemodynamic parameters remained stable intraoperatively. The recall of visual analogue scale scores during surgery was 2 [1–4] for median (range). The patients’ and surgeons’ satisfaction scores were 2 [1–4] and 1 [1–3] for median (range). No serious complications occurred.

**Conclusions:**

Combining ultrasound-guided CSSB and CCNB is a feasible, effective and safe technique for thyroidectomy.

**Trial registration:**

Current Controlled Trials ChiCTR-ONC-12002025. Registered 19 March 2012.

## Background

Recent descriptions regarding surgeries under local anesthesia have aroused a renewed interest in this approach for thyroid surgery [1–3]. However, a superficial cervical plexus block alone may be inadequate for thyroidectomy [4], even though fan-shaped injection of local anesthetic is employed. Although a deep block is effective, the successful performance of such a block can be difficult, and it is associated with the risk of intravascular or intrathecal injection, sympathetic block, pneumothorax, and hemidiaphragmatic paresis [5]. Compared with the superficial block, the deep technique is more than twice as likely to yield a serious life-threatening complication [6]. Additionally, the total dose of local anesthetic may approach toxic limits when deep and superficial blocks are combined.

Classically, the cervical plexus is considered to have two distributions, the superficial cutaneous and the deep motor nerves. The superficial branches, in their course towards the cervical tegument, perforate the investing layer of deep cervical fascia at the “nerve point of the neck” located in the middle of the posterior edge of sternocleidomastoid muscle (SCM). They are then distributed as four distinct nerves: the lesser occipital, greater auricular, transverse cervical, and supraclavicular nerves. The transverse cervical nerve and part of the medial supraclavicular nerve primarily innervate anterior region of neck [7]. Pandit *et al.* [8] reported that methylene blue dye did not spread beyond the subcutaneous tissue after subcutaneous injection (superficial to the investing fascia) in the cervical area. Therefore, we hypothesized that girdle-shaped injection of local anesthetic along SCM muscle in the subplatysma fat layer would effectively block the superficial cutaneous nerves and allow painless skin incision for thyroidectomy.

We recently identified a phenomenon in which the intraoperative incision and division of the thyroid capsule resulted in obvious discomfort and pain that was relieved by lidocaine spray. Anatomically, the thyroid gland has an inner true capsule which is thin and adheres closely to the thyroidal tissue [9]. External to this is a “false capsule” formed by the middle layer of the deep cervical fascia, which splits anterolaterally to ensheath the thyroid gland, thus forming the thyroid sheath [10]. In this fashion, the potential space called the capsule-sheath space is formed. It contains loose connective tissue, blood vessels, nerves and parathyroid gland.

Accordingly, we hypothesized that anesthetic deposited in this space would block the surface of thyroid and permeate directly into the parenchyma of the thyroid producing effective local anesthesia for surgical procedures involving the thyroid gland. Additionally, a subcutaneous injection along the sternocleidomastoid muscle (SCM) would also enhance effective local anesthesia for the initial skin incision and further contribute to a more ideal working environment for the surgeon. Therefore, we propose a new anesthetic technique termed ultrasound-guided capsule-sheath space block (CSSB) combined with anterior cervical cutaneous nerves block (CCNB) for thyroidectomy. After injection of contrast medium in healthy volunteers using this new technique, the pattern of distribution of contrast medium was imaged using computed tomographic scanning to provide the relevant anatomical basis for our hypothesis. Using this theoretical framework, the feasibility of the technique, its effect on intraoperative hemodynamic parameters, and documentation of analgesic effects and complications were assessed in 78 patients requiring thyroidectomy.

## Methods

### Imaging study

The study protocol was approved by the independent Ethics Committee at the First Affiliated Hospital of Wenzhou Medical University (Wenzhou, Zhejiang, China). It was also registered with Chinese Clinical Trial Registry under the number ChiCTR-ONC-12002025. Signed informed consent was obtained from all participants. In the imaging study, four females and one male healthy, ranging in age from 35 to 45 years of age, 150 to 171 cm in height, and weighing 41 to 65 kg, were enrolled in the imaging study. The subjects fasted from food for 6 hours and from liquid 4 hours before testing. Upon arrival to the computed tomographic examination room, standard ASA (American Society of Anesthesiologists) monitors were attached. Also, a peripheral intravenous access was established and Lactated Ringer’s solution was infused. The volunteers were sedated using 1–2 mg of intravenous midazolam and 100 μg of fentanyl prior to contrast medium injection.

An anesthesia nurse not involved in the study was asked to prepare four syringes each containing 2.5 mL Ultravist solution (Bayer HealthCare Pharmaceuticals Inc., Germany) diluted in normal saline solution to 10 mL. Ultrasound guidance was performed using a SonoSite MicroMaxx™ system (SonoSite Inc., Bothell, WA) with a 38-mm high frequency (6–13 MHz) linear array transducer.

**Figure 1:**
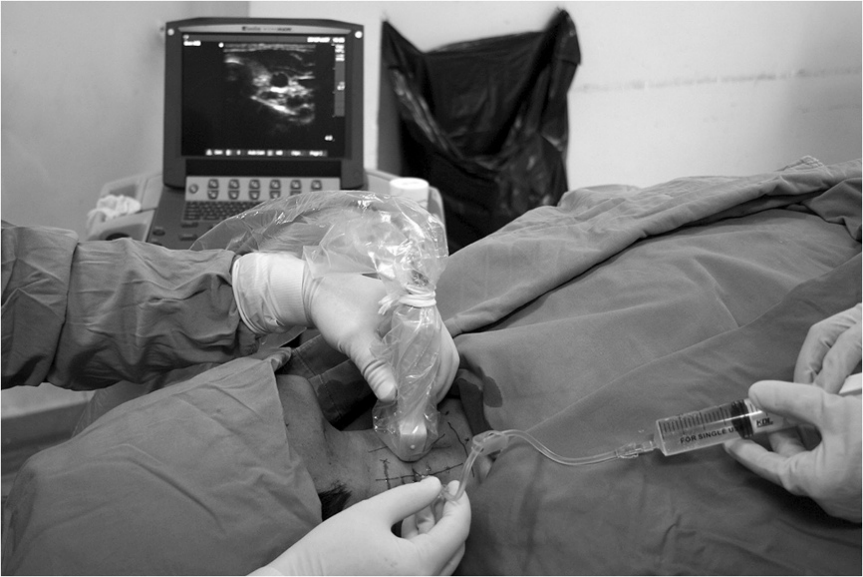
**Position of the ultrasound probe relative to the needle (needle-in-plane technique) for the ultrasound-guided capsule-sheath space block (CSSB) in a 45-yr-old male volunteer.**

**Figure 2:**
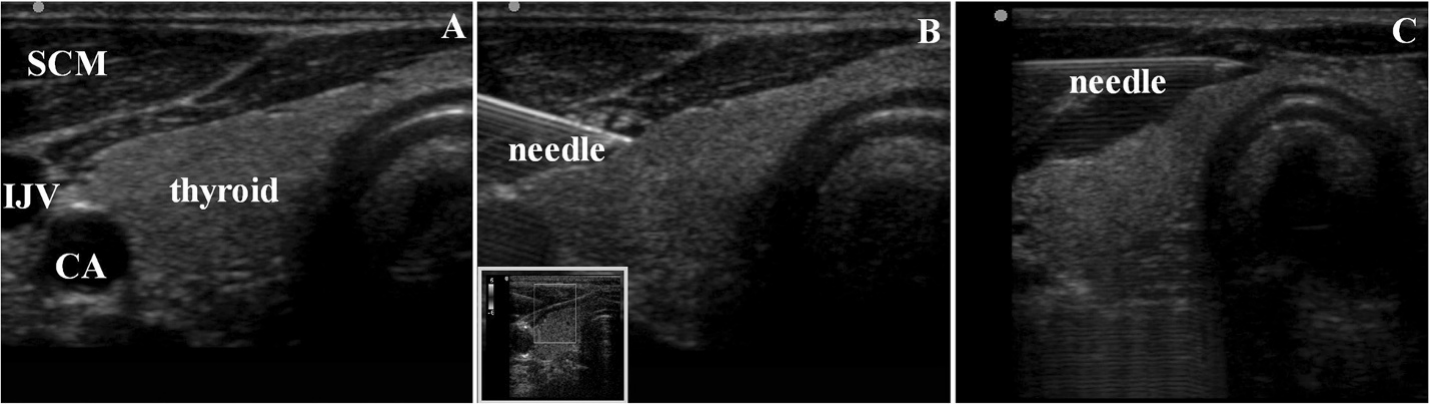
**The ultrasound-guided capsule-sheath space block (CSSB) in imaging study: ultrasonogram of the thyroid gland before and after local anesthetic injection. (A)** The juncture of internal jugular vein and thyroid gland was identified and placed in the center of the optimal image with minor adjustment of scanning planes; **(B)** In-plane view of a block needle inserted under color Doppler guidance; **(C)** Intermittent changes in needle direction to maintain a uniform distribution of contrast medium, especially at isthmus of thyroid. CA, carotid artery; IJV, internal jugular vein; LA, local anesthetics; SCM, sternocleidomastoid muscle.

The subjects were positioned supine with the head facing away from the side to be injected (Figure 1). The injection site was prepared in an aseptic manner, the operator used a sterile drape, gloves, mask and gown. The probe had a sterile covering. The ultrasound probe was placed in the transversal plane within anterior region of neck to visualize the unilateral thyroid gland in the transverse sectional view. The juncture of internal jugular vein and thyroid gland was then identified and was centered to produce the optimal image, with minor adjustment of scanning planes (Figure 2-A). The transverse sonograms clearly delineated medial trachea and the isthmus of thyroid, lateral internal jugular vein, and the superficial muscle group including the SCM muscle. In addition, Doppler imaging was used to identify the relevant arteries before needle insertion (Figure 2-B). A 50-mm, 21-gauge insulated block needle (HDC® Corporation, Milpitas, CA) was positioned at the outer (lateral) edge of the probe and advanced along the long axis using a needle-in-plane approach. The needle was identified passing through the skin, the superficial fascia, the sternocleidomastoid muscle, the omohyoid muscle, the false capsule, and into contact with the true capsule. Then 10 mL of contrast medium was deposited slowly into the capsule-sheath space with frequent aspirations over 1–2 min (Figure 2-C). Contrast medium spread at the time of injection was observed in real time. If the contrast medium did not reach some parts of the gland, the needle was repositioned once before depositing the remaining half of the contrast agent dose. Thereafter, contralateral blocking was performed with the same method.

**Figure 3:**
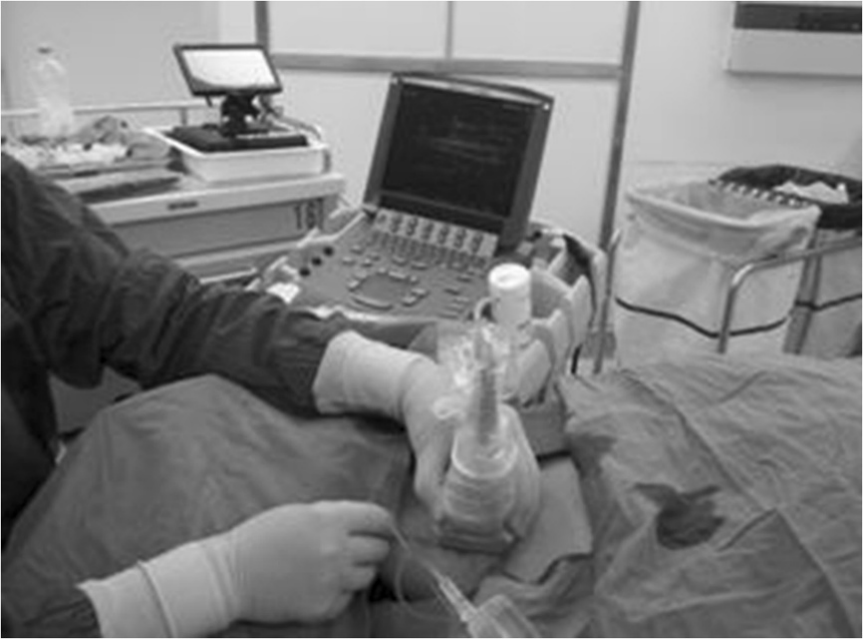
**Position of the ultrasound probe relative to the needle (needle-in-plane technique) for the ultrasound-guided anterior cervical cutaneous nerves block (CCNB).**

**Figure 4:**
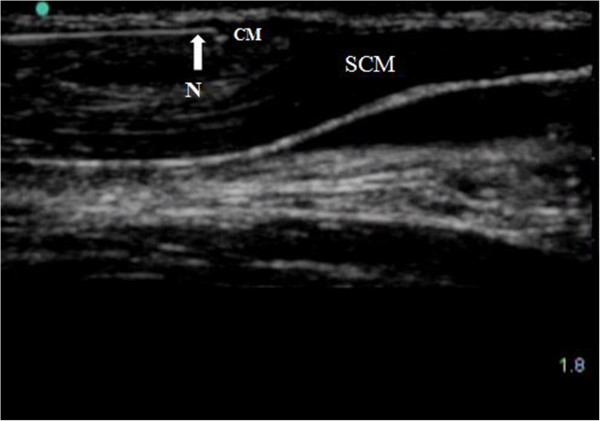
**The ultrasound-guided anterior cervical cutaneous nerve block. N, needle; CM, contrast medium; SCM, sternocleidomastoid muscle.**

The subjects were placed in a supine position with the head turned away from the side to be blocked (Figure 3), and then the SCM muscle was identified by slight head elevation. Landmarks, mastoid process, and clavicle heads of the SCM were marked with a pen and then a line was drawn in the midline along the SCM, as well as the midpoint of the line. Under ultrasound guidance (in plane technique) (Figure 4), 10 mL of contrast medium was injected subcutaneously along the line from the midpoint to the clavicular heads of the SCM. A girdle-shaped picchu with a width of 1.0 cm was raised from the cricoid cartilage to supraclavicular region. To complete the bilateral injections the sequence was repeated on the other side.

### CT contrast examination

A computed tomography (CT) scan was conducted 10 min after injection to examine the pattern of contrast medium spread.

### Clinical case series

**Figure 5:**
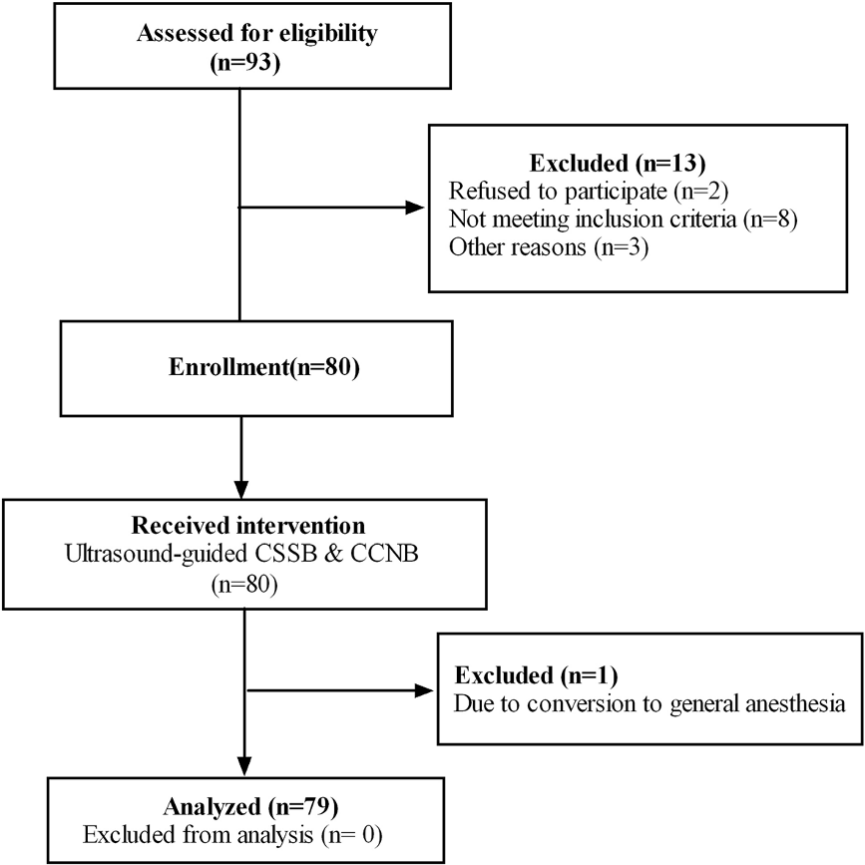
**Flow diagram of patients throughout the trial. capsule-sheath space block (CSSB), anterior cervical cutaneous nerves block (CCNB).**

The study protocol was approved by the independent Ethics Committee at the First Affiliated Hospital of Wenzhou Medical University (Wenzhou, Zhejiang, China). It was also registered with Chinese Clinical Trial Registry under the number ChiCTR-ONC-12002025. Signed informed consent was obtained from all participants. Seventy-eight patients scheduled for elective thyroidectomy were consecutively enrolled into the study between November 1, 2010 and February 20, 2011. The eligibility criteria included patients age 20–65 years, American Society of Anesthesiologists physical status I–II, simple or multinodular goiter with a goiter diameter less than 2 cm, and euthyroidism after thyroid function tests (TSH, free triiodothyronine and thyroxine). Exclusion criteria included a nodule diameter larger than 2 cm, substernal goiter, contraindication or patient refusal to regional anesthesia, a history of difficult intubation, probable cervical lymph node dissection for enlargement of lymph nodes diagnosed by preoperative sonography, pulmonary or cardiac risk factors, language barrier, and a previous history of cervical surgery. A flow diagram of patients recruited for the study is depicted in Figure 5.

### Pre-anaesthetic preparation

Two 10-mL syringes fully containing equal volumes of 0.375% ropivacaine (Naropin® 10 mg/mL, AstraZeneca-AB, Sweden) and 1.0% lidocaine (Cheng Yi Pharmaceutical Co., Ltd., Zhengjiang, China) were prepared for CCNB; another two 10-mL syringes fully containing 0.375% ropivacaine were prepared for bilateral CSSB.

All subjects fasted at least 6 hours from food and 4 hours from liquids before their surgeries. In addition, they were instructed in regard to the study protocol and VAS. Upon arrival to the operating room all patients received intravenous access and standard ASA monitoring. This was continued until discharge from the postanesthesia care unit. The patients were adequately sedated prior to block placement using the same sedative as those used in initial imaging study.

### Block performance

Ultrasound-guided CSSB and CCNB were performed as previously described for the contrast medium injection in the imaging study. In cases of total thyroidectomy or bilateral subtotal thyroidectomy, a bilateral block was performed. For patients undergoing lobectomy and isthmusectomy, a unilateral block was performed and an additional 3–5 mL of local anesthetic solution was injected at the thyroid isthmus.

### Adjuvant drug

Intermittent treatments with the aforementioned sedative were given intraoperatively for all patients to maintain a desired level of sedation. Inadequate block to the surgical area was supplemented with 10 mg ketamine. If the patient still experienced pain despite supplementation, it was defined as a local anesthetic failure and managed with conversion to general anesthesia. Respectively, esmolol and urapidil hydrochloride were given intravenously when the heart rate or the systolic blood pressure increased more than 20% as compared with the baseline value. Additionally, 5–10 mg of intravenous ephedrine was administered if the systolic blood pressure decreased to 80 mmHg or when it decreased more than 30% as compared with the baseline value. Atropine was administered if the heart rate declined to 50 beats/min. For postoperative pain management, parecoxib (Dynastat®, Pfizer Inc, NY) 40 mg was given on the first evening after surgery to all patients. On the ward, 50 mg of meperidine was given intramuscularly if pain score was 4 or higher at rest.

Thyroid surgery was performed according to a standardized procedure by the same surgeons and the anesthetic was administered by senior consultant anesthetists. Data collection and statistical analysis were performed by two other junior anesthetists.

### Outcome measure

We recorded the mean arterial blood pressure (MAP), heart rate (HR), and saturation of blood oxygen (SpO_2_) before the administration of local anesthesia, and at 5, 10, 20, 30, 40, 50, and 60 min after the nerve block was placed. Also monitored were the block execution time (the interval between skin sterilization and the needle removal after local anesthetic injection), the sensory onset of CCNB (the interval between the end of injection and analgesia), the operating time (the time elapsed from incision of skin to closure), the operative methods, the pattern of local anesthetic spread, postblock complications, including postoperative nausea and vomiting, dizziness, headache, hoarseness, and respiratory embarrassment. A 11-point verbal pain scale was administered (0, no pain; 10, worst pain imaginable) to the patients [11]. On arrival to the postanesthesia care unit, the patients were asked to recall how much pain they experienced and their degree of satisfaction with their surgical experience. Immediately after surgery, the surgeons were questioned as to their satisfaction with the operating conditions (i.e. ‘very satisfied’, ‘satisfied’, ‘average’, ‘poor’, and ‘very poor’) [12]. Postoperative pain was also assessed at 4 h and 8 h after surgery.

### Ropivacaine measurement

Specimens from nine patients were resected intraoperatively for ropivacaine determination and were rinsed to remove any residual blood before being submitted for analysis. After resection of nodules, the remaining normal gland tissue was minced into 3 × 3 mm fragments and then stored at -70°C (-94°F) until the analysis was performed. Thyroidal tissue content of ropivacaine was analyzed in these nine patients using high performance liquid chromatography/mass spectrometry (HPLC/MS) [13].

### Statistical analysis

Data with normal distribution are presented as mean ± standard deviation. The other quantitative variables are reported as median and range because their potentially skewed distribution. Mean arterial pressure (MAP), heart rate (HR) peripheral capillary oxygen saturation (SpO_2_) were analyzed by using a repeated-measures analysis of variance followed by multiple comparisons with Bonferroni adjustments to evaluate significant changes from pre-anesthesia values. To ensure that all patients included in the study could be analyzed as to the clinical efficacy of the intervention, the last-observation-carry-forward principle was applied and the missing values for the data of MAP, HR, and SpO_2_ were replaced by the last value available for the patients whose operative time was less than 60 mins. All statistical analysis was performed with SPSS for Windows (version 14.0; SPSS, Chicago, IL). Statistical significance was considered as *P* <0.05.

## Results

### Imaging study

**Figure 6:**
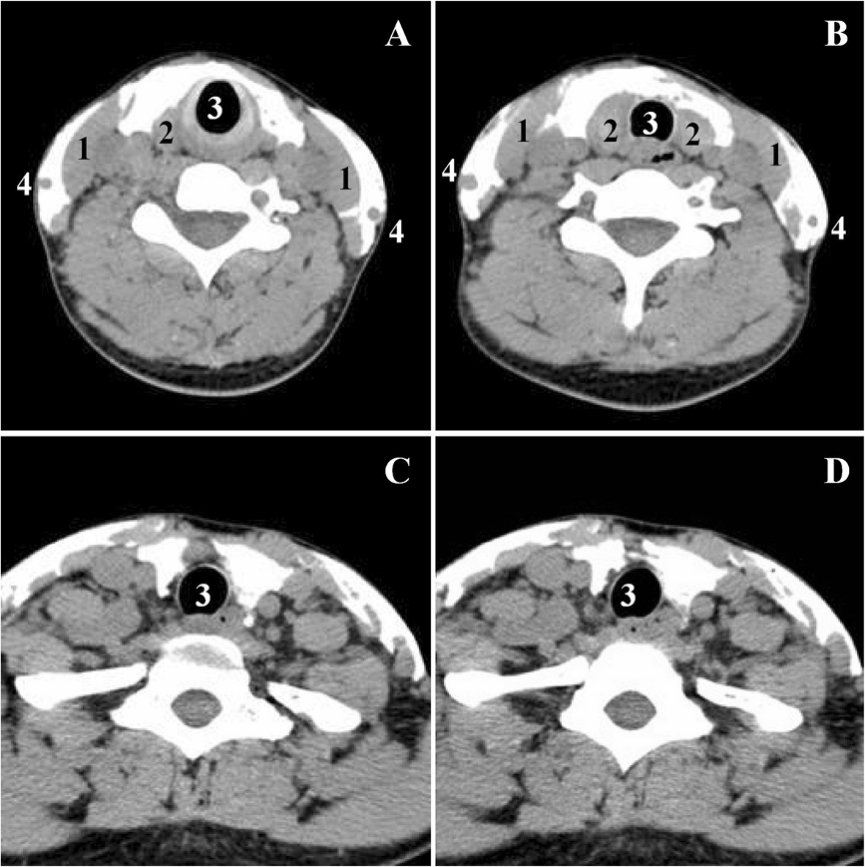
**Computed tomography (CT) images of spreading patterns of the contrast medium in ultrasound-guided capsule-sheath space block (CSSB) combined with anterior cervical cutaneous nerves block (CCNB).** CT scan was respectively obtained at the level of upper pole **(A)**, middle of the lobes **(B)**, lower pole **(C)**, and cervical region VI **(D)** in a 35-yr-old female volunteer. It demonstrated that the injectate spread along bilateral SCM muscles in superficial compartment. Likewise, the injectate in capsule-sheath space could spread up to the upper pole and down to the lower pole and even lower region VI. Furthermore, contrast medium reached the posterior aspect of the two poles. 1, sternocleidomastoid muscle; 2, thyroid gland; 3, trachea; 4, external jugular vein.

The CT imaging distinctly demonstrated the spread of the contrast medium along the SCM muscles bilaterally in the superficial compartment. Likewise, the injectate placed the in capsule-sheath space spread up to the upper pole and down to the lower pole, and even lower to region VI. Furthermore, contrast medium reached the posterior aspect of the poles (Figure 6).

### Clinical case series

Ninety-three patients were considered eligible for inclusion in this study. Two patients refused to participate and eight did not meet the inclusion criteria due to nodule diameter larger than 2 cm. Three participants were excluded by the researchers for the equipment constraints. Two other patients were excluded because it was necessary to convert to general anesthesia because one patient required a bilateral lymph node dissection and another patient felt pain during operation. Consequently, 78 patients (67 women and 11 men) completed the study protocol (Figure 5). The patients’ ages ranged from 19 to 65 years, with a mean ± SD of 44 ± 10 years. Mean ± SD height and weight were 160 ± 5 cm and 61 ± 11 kg, respectively. The block execution time was 11.2 ± 1.8 min, and the sensory onset of CCNB was 2.2 ± 0.6 min. A limited collar incision was made for all patients. Hemostasis was achieved using ultrasonic scissors. The procedures performed included 29 bilateral subtotal thyroidectomies, 18 left and 23 right total lobectomies, and 8 total thyroidectomies. In one case, a total thyroidectomy was accompanied by bilateral lymph node dissection (see above). The mean operating time was 60 ± 25 min. The cytological/histological identification of the nodules was determined during the operation or postoperativeley (Table 1).

**Table 1:** **Patient demographics and surgical data of clinical case series (n = 78)**

Age (yr)	44 ± 10
Body weight (kg)	61 ± 11
Height (cm)	160 ± 5
Male: female ratio	11:67
Surgery (n)	
Lobectomies	41
Subtotal thyroidectomy	29
Total thyroidectomy	8
Block execution time (min)	11.2 ± 1.8
Sensory onset time of CCNB (min)	2.2 ± 0.6
Duration of surgery (min)	60 ± 25
Cytological/histological (n)	
Simple goiter	8
Thyroid adenoma	35
Papillary thyroid carcinoma	27
Medullary thyroid carcinoma	5
Anaplastic thyroid carcinoma	3

Values are given as mean ± standard deviation. CCNB = anterior cervical cutaneous nerves block.

### Typical ultrasonogram

**Figure 7:**
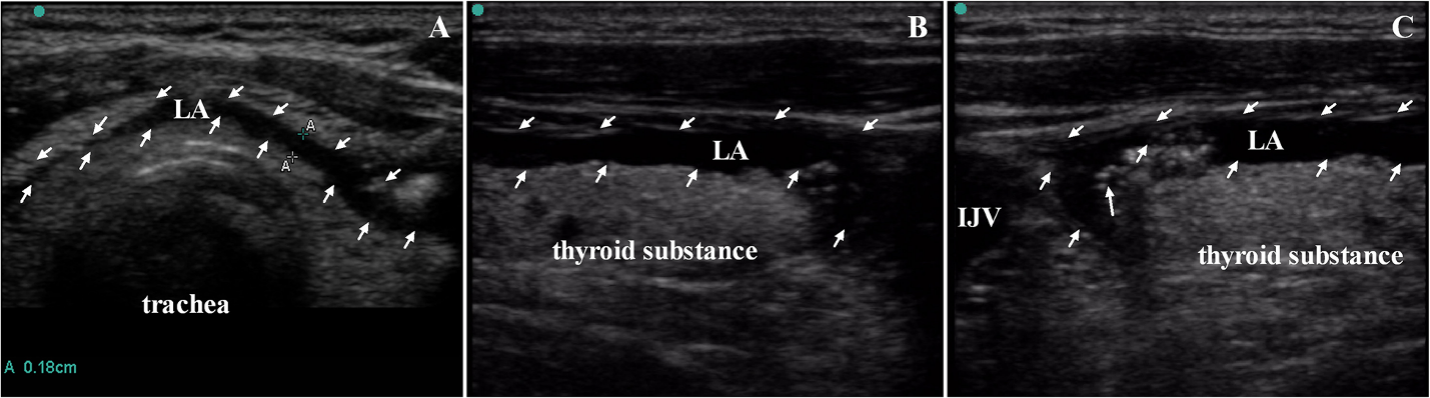
**Typical ultrasonogram of the distribution of local anesthetics after ultrasound-guided capsule-sheath space block (CSSB) was performed in a 38-yr-old female patient. (A)** Sonogram of the thyroid gland in the transverse plane showing the uniform distribution of local anesthetics solution thickness 0.18 cm; **(B)** Sonogram of upper pole of the thyroid gland in the longitudinal plane showing the uniform local anesthetics solution around thyroid; **(C)** Sonogram of lower pole of the thyroid gland in the longitudinal plane. IJV, internal jugular vein; LA, local anesthetics; SCM, sternocleidomastoid muscle.

**Figure 8:**
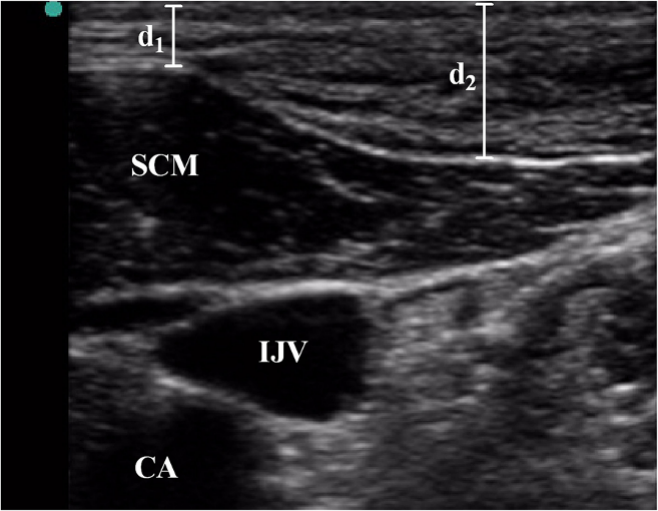
**Typical ultrasonogram of the distribution of local anesthetics after the anterior cervical cutaneous nerves block (CCNB) was performed in a 41-yr-old female patient.** IJV, internal jugular vein; CA, carotid artery; SCM, sternocleidomastoid muscle; d1/d2, depth of the distribution of local anesthetics.

For all patients, ultrasonography enabled visualization of the distribution of local anesthetic around the targeted lobes including the upper and lower poles, as well as the isthmus of the thyroid gland, just below the false capsule after ultrasound-guided CSSB (Figure 7). Similarly, spread of the injected local anesthetic solution after CCNB is illustrated in Figure 8. Local anesthetic solution was identified in the supraclavicular region within superficial fascia, including subcutaneous tissue and platysma.

### Quality of the blocks

In general, light conscious sedation was the objective of our anesthetic technique. During the block, monitoring, and line insertion, patients were sedated with midazolam and fentanyl (Table 2). During surgery, we made use of pharmacological interventions to ensure patients’ comfort and safety (Table 3). Pain scores, and patient and surgeon satisfaction scores during the operative period are presented in Table 4. Pain scores at 4 h and 8 h after surgery were 2 [range: 0–3] and 2 [range: 1–4]. During the resection of the affected isthmus, one patient experienced intraoperative pain and then was treated with intravenous ketamine, but was still uncomfortable and was converted to general anesthesia. In this case, subsequent retrospective analysis of ultrasound images further verified that the local anesthetic administered did not reach the isthmus or the upper pole.

**Table 2:** **Preoperative pharmacological interventions (n = 78)**

Patients requiring midazolam, n preoperatively	78 (100%)
Total amount of midazolam, mg	1 [0.5–2]
Patients requiring fentanyl, n	78 (100%)
Total amount of fentanyl, μg	75 [25–100]

Values are number (%) or median (range).

**Table 3:** **Intraoperative pharmacological interventions (n = 78)**

Patients requiring midazolam, n preoperatively	47 (60.3%)
Total amount of midazolam, mg	0.5 [0–2]
Patients requiring fentanyl, n	31 (39.7%)
Total amount of fentanyl, μg	0 [0–100]
Patients requiring ketaminel, n	5 (6.3%)
Total amount of ketamine, mg	0 [0–10]
Patients requiring esmolol, n	11 (14.1%)
Total amount of esmolol, mg	0 [0–100]
Patients requiring urapidil, n	7 (9%)
Total amount of urapidil, mg	0 [0–50]
Patients requiring atropine, n	0
Patients requiring ephedrine, n	0

Values are number (%) or median (range).

**Table 4:** **Regional block quality (n = 78)**

Pain score preoperatively	2 [1-4]
Patient satisfaction score	2 [1-3]
Surgeon satisfaction score	1 [1-3]

Patient and surgeon satisfaction score was measured on a 5-point scale (1, very satisfied; 2, satisfied; 3, average; 4, poor; and 5, very poor). All data are presented as median (range).

### Intraoperative hemodynamic values

**Figure 9:**
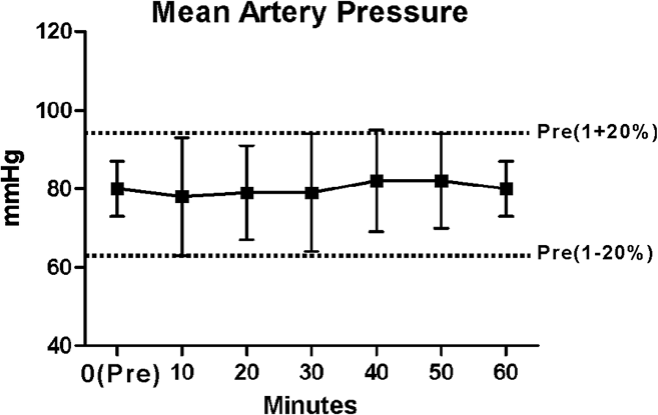
**Mean artery pressure** ***versus*** **time for 78 patients completed the study protocol.** The mean artery pressure at 10, 20, 30, 40, 50 and 60 mins post anaesthesia were no different with pre-anaesthesia values. All of mean values did not exceed ± 20% of the pre-anesthesia mean values, which were deemed clinically acceptable. Data are mean and error bars SD.

**Figure 10:**
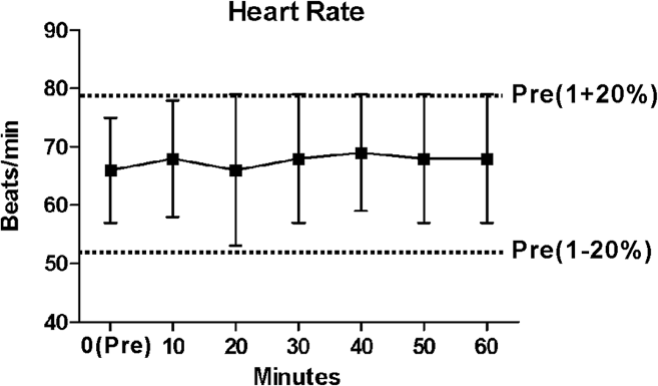
**Heart rate** ***versus*** **time for 78 patients completed the study protocol.** No significant changes from pre-anesthesia values were observed, and all of mean values were within ± 20% of the pre-anesthesia mean values. Data are mean and error bars SD.

Overall, the MAP, HR, and SpO_2_ were stable intraoperatively. MAPs at 10, 20, 30, 40, 50 and 60 mins post anaesthesia did not differ from pre-anesthesia values (at 0 min). All MAPs did not exceed ± 20% of the MAPs before block performance, and were within a “clinically acceptable” range (Figure 9). With regard to HR, no significant changes from values before block performance were observed (Figure 10). One patient, who intravenously received 2 mg of midazolam and 100 μg of fentanyl, experienced an SpO_2_ level of less than or equal to 94%. The episode of sedation-associated hypoxia was reversed by supplemental oxygen. There were no reports of respiratory depression, hypotension or bradycardia throughout the operation.

### Postblock complications

Four patients developed postoperative nausea, among which one patient had vomiting. Four patients experienced dizziness and two suffered headache. Additionally, there was one case of paroxysmal coughing. No hoarseness or respiratory embarrassment was noted, and no hematoma or infection was reported.

### Ropivacaine measurement

Ropivacaine was measurable in all nine of the thyroid tissue samples. The mean concentration of ropivacaine was 10.5 ± 5.4 μg/g and the range is 4.1-18.9 μg/g.

## Discussion

The results of the imaging study with contrast medium demonstrated that the subcutaneous injectate that was infiltrated along the SCM could uniformly distributed within the superficial fascia. Likewise, the injectate in capsule-sheath space spread up to the upper pole and down to the lower pole or even lower portion of region VI. In our subsequent clinical evaluation, good surgical anesthesia was obtained by using the same injection technique.

A superficial cervical plexus block alone may not be satisfactory for thyroidectomy because the strap muscles and visceral structures including part of the tracheal fascia and the surgical capsule may not be adequately anesthetized by this technique [4]. In addition, the inappropriate distance between the needle tip and the target nerve with landmark-based localization and the variations of nerve point of neck may also contribute to the inadequacy of anesthesia [14].

Stoneham *et al.* [15] have successfully used a two-point injection with 20 ml of 0.375% bupivacaine that allowed painless procedures to be performed. The injection target layers and spread in Stoneham *et al.* was the same as ours. Nevertheless, our girdle-shaped injection of less local anesthetic volume (5 ml on each side) may theoretically produce a more complete blockade of cutaneous nerves than a subcutaneous “bleb” of anesthetic solution. We agree with Pandit *et al.* [8] that an injection superficial to investing layer had a lower or null risk of anesthesia of deep nerves and thus eliminated the possibility of diaphragmatic hemiparesis and respiratory difficulty.

To obtain excellent exposure of the thyroid gland, all patients in our study received routine separation of the strap muscles, after which the cricothyroid space was opened, thus exposing the middle layer of cervical fascia [10, 16]. Following the incision and division of the thyroid capsule, the spread of local anesthetic was confirmed under direct vision. The thyroid was infiltrated by anesthetic solution anteriorly and anterolaterally. The distribution of solution around the upper and lower poles was also identified. Thus we inferred that anesthetic injection into the capsule-sheath space would block autonomic fibers to reduce patients’ discomfort. We also detected ropivacaine in all thyroidal tissue samples after injection of anesthetic in the capsule-sheath space, which strongly suggests that the local anesthetic deposited in this space entered the parenchyma of thyroid gland. Cunliffe *et al.* [17] demonstrated that most nerves enter the gland either as vascular nerve bundles and plexuses or follicular nerves, the former were associated closely with the arteries and supplied fibers to the vascular plexuses while the latter primarily arose from the vascular nerve bundles, and eventually entered the parenchyma as the secretomotor supply. Accordingly, one possible explanation of our novel finding is that the inner true capsule may be “porous” and allow spread of the ropivacaine molecule, and this “porousness” might be associated with the site at which the vessels and nerves pierce the inner capsule to enter the thyroid parenchyma. The fact that the complex surgical maneuvers required during these surgeries, including those associated with the dorsal surface could be successfully performed by the surgical team, as well as the detection of ropivacaine in all nine thyroid tissue samples further suggests that the most likely reason for this effective surgical anesthesia was that the ropivacaine crossed the inner capsule and successfully blocked the intrinsic nerves of thyroid gland.

Outcomes with regard to postblock complications, quality of the blockade, and duration of surgery were substantiated based on the lack of adverse hemodynamic events under CSSB combined with CCNB or complications in the perioperative period. Although the recurrent laryngeal nerve lays within the pretracheal fascia, it was not in the same layer as the distribution of ropivacaine because the left aspect was situated deep in the tracheoesophageal sulcus, and the right aspect was usually distant from the trachea [18], thereby minimizing the chance of any complications relating to the recurrent laryngeal nerve in any patient.

With respect to the performance of CSSB, the following points are of paramount importance: (1) the space between the true and false capsules is traversed by the larger arterial and venous branches. Doppler ultrasound guidance can effectively assist in avoiding vascular damage; (2) in-plane needling technique is recommended to facilitate successful needle tip localization. Needle advancement should be halted once the needle tip visibility is lost at any time, particularly when it is in close proximity to vessels or the true capsule; (3) Since the capsule-sheath space is narrow, high-ultrasound frequency is needed to generate enough resolution to determine whether or not capsule was pierced; (4) the local anesthetic solution usually spreads downward from the middle of the gland lobe towards the lower pole after needle insertion in a lateral direction, especially in the case of a large gland. Therefore, intermittent changes in needle direction are usually necessary to maintain a uniform distribution of anesthetic solution; (5) needle insertion depth should be carefully controlled during the execution of CCNB. We suggest injecting local anesthetic solution superficial to the investing layer in order to avoid an injection into the SCM. The uniform distribution of local anesthetic solution is essential to successful blockade, and (6) careful attention to injection technique and appropriate post-injection monitoring are essential.

In the present study, we propose a new local anesthetic technique. We confirmed the technique’s feasibility (in healthy volunteers) through the use of computed tomography in order to demonstrate the appropriate spread of the local anesthetic. After its feasibility was confirmed the applicability/efficacy for its use in thyroidectomy was verified in 78 patients. Its use has an advantage of limiting postoperative pain and its adverse effects. Also, its use may avoid sore throat from endotracheal intubation, which is commonly associated with general anesthesia. Although one may be able to argue that it may have an advantage in those patients with an anticipated or unanticipated difficult airway, caution should be exercised any time when a block is applied to such patients. In our study, electrophysiological recurrent laryngeal nerve monitoring was available during thyroid surgery.

A limitation of this pilot study was the lack of control group, thereby making it difficult to evaluate the relative superiorities such as the anesthetic effect, the total cost of anesthesia, the complications, and patient satisfaction when compared with conventional cervical plexus block or general anesthesia. Moreover, the ideal volume and concentration of local anesthetic for CSSB, as well as pharmacokinetic analysis have still to be determined by future studies.

## Conclusion

Combining capsule-sheath space block and anterior cervical cutaneous nerve block under ultrasound guidance is feasible and shows safety and efficacy. It is associated with fast onset, satisfactory analgesia and without serous complications. However, larger multi-institutional studies need to be performed to replicate the results presented here.

## Abbreviations

CSSB: Capsule-sheath space block
CCNB: Cervical cutaneous nerve block
SCM: Sternocleidomastoid muscle
MAP: Mean arterial pressure
HR: Heart rate
SpO_2_: Peripheral capillary oxygen saturation.
